# Norovirus Surveillance among Callers to Foodborne Illness Complaint Hotline, Minnesota, USA, 2011–2013

**DOI:** 10.3201/eid1908.130462

**Published:** 2013-08

**Authors:** Amy A. Saupe, Dawn Kaehler, Elizabeth A. Cebelinski, Brian Nefzger, Aron J. Hall, Kirk E. Smith

**Affiliations:** Minnesota Department of Health, St. Paul, Minnesota, USA (A.A. Saupe, D. Kaehler, E.A. Cebelinski, B. Nefzger, K.E. Smith);; Centers for Disease Control and Prevention, Atlanta, Georgia, USA (A.J. Hall)

**Keywords:** norovirus, hotlines, genotype, incidence, gastroenteritis, Caliciviridae, diarrhea, public health, molecular epidemiology, Minnesota, United States, viruses, enteric infections

## Abstract

Norovirus is the leading cause of foodborne disease in the United States. During October 2011–January 2013, we conducted surveillance for norovirus infection in Minnesota among callers to a complaint-based foodborne illness hotline who reported diarrhea or vomiting. Of 241 complainants tested, 127 (52.7%) were positive for norovirus.

Norovirus is the leading cause of foodborne disease and sporadic and outbreak-associated acute gastroenteritis in the United States (*1**,**2*), accounting for 21 million illnesses, 70,000 hospitalizations, and 800 deaths each year (*3*). Norovirus is not routinely tested for in clinical settings because detection requires molecular methods typically available only in public health and research laboratories. Therefore, characterization of norovirus epidemiology has been primarily through analysis of outbreak data.

Consistent with national trends (*4*), most foodborne disease outbreaks identified in Minnesota are caused by norovirus. In addition, most foodborne outbreaks in Minnesota, including virtually all norovirus outbreaks, are identified through a centralized foodborne illness complaint hotline system, operated by the Minnesota Department of Health (MDH) (*5**,**6*). However, most calls to the hotline represent sporadic (i.e., non–outbreak-associated) illness; only ≈7% of complaints are associated with known outbreaks (*5*). Systematic testing of hotline callers to determine illness etiology has not previously been conducted.

In this study, we conducted surveillance for norovirus among hotline callers. Our objectives were to characterize the role of norovirus as a cause of gastroenteritis in hotline callers and to describe trends in norovirus infection in this population as an indicator for norovirus activity in Minnesota.

## The Study

The MDH foodborne illness complaint system has been described in detail (*5**,**6*). From October 1, 2011, through January 31, 2013, eligible hotline callers (complainants) were asked to submit a self-collected fecal sample to the MDH Public Health Laboratory (PHL). Complainants were eligible to submit a stool sample on the basis of reported symptoms (>3 loose stools in 24 hours or vomiting [symptom eligibility]) and other criteria, including timeliness of complaint (Technical Appendix). If the original complainant was not eligible for or refused testing, another ill person reported in the complaint (co-complainant) was asked to submit a stool sample, if eligible. Only 1 stool sample per complaint was used in analyses. This surveillance effort was exempted from review by the MDH Institutional Review Board.

Specimen vials were refrigerated on receipt at the MDH PHL and batch tested weekly. Detection and characterization of norovirus strains were performed by using the Centers for Disease Control and Prevention CaliciNet methods (*7*). Briefly, detection of norovirus genogroups I and II was performed by duplex real-time reverse transcription PCR. Genotypes were determined by sequence analysis of the viral capsid gene and phylogenetic comparison with CaliciNet reference strains.

On the basis of the known winter seasonality of norovirus outbreaks (*8*), norovirus season was defined as October–March and the off-season as April–September. Data analysis was performed by using SAS version 9.2 software (SAS Institute Inc., Cary, NC, USA).

During October 2011–January 2013, the Minnesota foodborne illness hotline received 1,060 calls (median 60 calls/mo) (Table 1). The mean number of monthly calls to the hotline was greater during the norovirus season than during the off-season (73.6 vs. 54.0; p = 0.025). A total of 633 (59.7%) complainants or co-complainants met the eligibility requirements for stool sample submission; of these, 241 (38.1%) submitted a sample that was included in analyses.

**Table 1:** Demographics, signs and symptoms, and epidemiologic characteristics for callers to foodborne illness hotline, Minnesota, USA, October 2011–January 2013*

Characteristic	All symptom-eligible complainants				Complainants tested for norovirus		
	Tested, n = 241	Not tested,† n = 700	p value‡		Positive, n = 127	Negative, n = 114	p value‡
Age, y (range)	44 (0–88)	43 (1–91)	0.62		44 (1–88)	44 (0–88)	0.49
Duration, h (range)	30.80 (0.25–205.30)	29.0 (0.1–302.0)	0.63		36.0 (9.5–121.0)	18.00 (0.25–205.30)	**0.002**
Female sex	138 (57.3)	405 (58.1)	0.82		71 (55.9)	67 (58.8)	0.65
Signs and symptoms							
Diarrhea	193 (81.4)	521 (81.0)	0.30		103 (83.0)	90 (79.6)	0.50
Vomiting	183 (76.3)	513 (74.0)	0.50		110 (87.3)	73 (64.0)	**<0.001**
Bloody stools	9 (4.5)	23 (4.3)	0.94		2 (2.0)	7 (7.0)	0.09
Fever	81 (45.0)	152 (30.6)	**<0.001**		46 (52.9)	35 (37.6)	**0.04**
Onset during norovirus season§	172 (71.4)	486 (69.4)	0.57		108 (85.0)	64 (56.1)	**<0.001**
Health care visit	14 (6.5)	91 (13.5)	**0.006**		6 (5.6)	8 (7.5)	0.58
Outbreak associated¶	30 (12.4)	53 (7.6)	–		24 (18.9)	6 (5.3)	–

*Values are no. (%) except as indicated. Symptom-eligible complainants were callers who reported diarrhea (>3 loose stools in 24 h) or vomiting. Denominators vary because of missing data. **Boldface** indicates significance. †Includes all complainants eligible to submit a sample on the basis of symptom profile alone (symptom eligibility) as well as complainants not eligible to submit a stool sample for testing based on other eligibility criteria. ‡χ^2^ test was used for categorical variables; Fisher exact test used when expected cell frequencies were <5; Wilcoxon 2-sample test was used for comparison of medians. §Season defined as October–March. ¶One complainant per outbreak identified through the hotline was included in analyses, if otherwise eligible.

**Figure 1:**
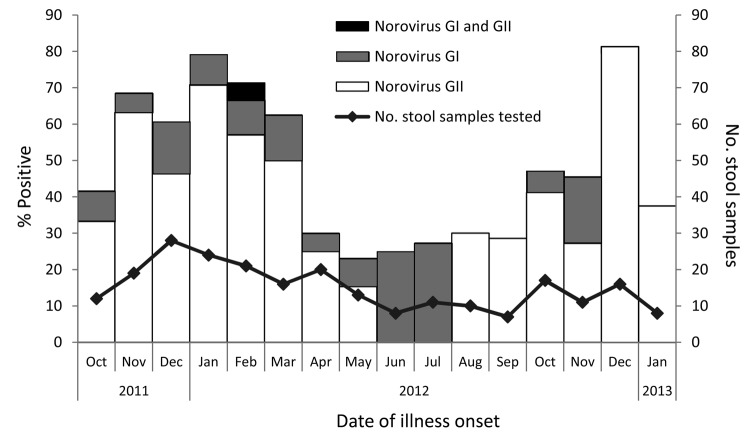
Percentage of stool samples submitted by callers to foodborne illness hotline that were positive for norovirus, by month of illness onset and genogroup, Minnesota, USA, October 2011–January 2013.

Of the 241 stool samples, 127 (52.7%) were positive for norovirus: 22 (17.3%) for genogroup I, 104 (81.9%) for genogroup II, and 1 for genogroups I and II (Table 1; Figure 1). The monthly percentage of norovirus-positive samples varied from 23.1% in May 2012 to 81.3% in December 2012 (Table 1; Figure 1). Complainants who called during the norovirus season were more likely to test positive for norovirus than were those who called during the off-season (62.8% vs. 27.5%; p<0.001) (Table 2). Norovirus-positive complainants were more likely than norovirus-negative complainants to report vomiting (87.3% vs. 64.9%; p<0.001) and fever (52.9% vs. 36.2%; p = 0.049) and to have longer illness duration (median 36 vs. 18 hours; p<0.001) (Table 2).

**Table 2:** Results for testing of stool samples submitted by callers to foodborne illness hotline, by month of complainant illness onset, Minnesota, October 2011–January 2013

Year and month	Total no. complainants	No. (%) eligible to submit sample*	No. (%) tested	No. (%) positive for		
				Any norovirus	Norovirus genogroup I†	Norovirus genogroup II†
2011						
Oct	48	28 (58.3)	12 (42.9)	5 (41.7)	1 (20.0)	4 (80.0)
Nov	52	37 (71.2)	19 (51.4)	13 (68.4)	1 (7.7)	12 (92.3)
Dec	117	70 (59.8)	28 (40.0)	17 (60.7)	4 (23.5)	13 (76.5)
2012						
Jan	96	62 (64.6)	24 (38.7)	19 (79.2)	2 (10.5)	17 (89.5)
Feb‡	67	46 (68.7)	21 (45.7)	15 (71.4)	2 (13.3)	12 (80.0)
Mar	79	40 (50.6)	16 (40.0)	10 (62.5)	2 (20.0)	8 (80.0)
Apr	57	34 (59.6)	20 (58.8)	6 (30.0)	1 (16.7)	5 (83.3)
May	54	38 (70.4)	13 (34.2)	3 (23.1)	1 (33.3)	2 (66.7)
Jun	51	29 (56.9)	8 (27.6)	2 (25.0)	2 (100.0)	0
Jul	54	26 (48.1)	11 (42.3)	3 (27.3)	3 (100.0)	0
Aug	62	35 (56.5)	10 (28.6)	3 (30.0)	0	3 (100.0)
Sep	46	30 (65.2)	7 (23.3)	2 (28.6)	0	2 (100.0)
Oct	80	47 (58.8)	17 (36.2)	8 (47.1)	1 (12.5)	7 (87.5)
Nov	79	46 (58.2)	11 (23.9)	5 (45.5)	2 (40.0)	3 (60.0)
Dec	76	44 (57.9)	16 (36.4)	13 (81.3)	0	13 (100.0)
2013 Jan	42	21 (50.0)	8 (38.1)	3 (37.5)	0	3 (100.0)
Total	1,060	633 (59.7)	241 (38.1)	127 (52.7)	22 (17.3)	104 (81.9)

*All samples tested at Minnesota Department of Health (MDH) Public Health Laboratory. Eligibility criteria: Minnesota residency, symptom eligibility (>3 loose stools in 24 h or vomiting), complaint received <4 d after vomiting/diarrhea onset or <2 d after vomiting/diarrhea recovery, and complainant interviewed by MDH staff. Completed complaints forwarded to MDH by local jurisdictions were excluded. †Of those positive for norovirus. ‡One February complainant was positive for genogroups (not included in individual genogroup totals).

**Figure 2:**
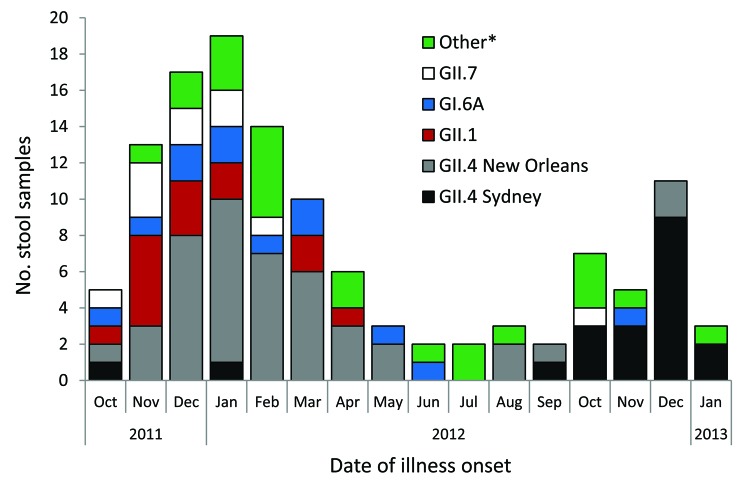
Norovirus genotypes identified in stool samples submitted by norovirus-positive callers to the foodborne illness hotline, Minnesota, USA, October 2011–January 2013. *Other genotypes identified: GII.4 Minerva, GI.3B, GII.3, GI.2, GI.7, GII.12, GI.4, GI.5, GII.6, GII.8

The most common genotypes among the 122 norovirus-positive specimens that could be sequenced were GII.4 New Orleans (44, 36.1%), GII.4 Sydney (20, 16.4%), GII.1 (14, 11.5%), GI.6 (12, 9.8%), and GII.7 (10, 8.2%) (Figure 2). GII.4 New Orleans was predominant during the 2011–2012 norovirus season, and GII.4 Sydney was most common during the first 4 months of the 2012–2013 norovirus season (Figure 2).

## Conclusions

This study highlights the predominant role of norovirus infections among callers to a foodborne illness complaint hotline in Minnesota. Call volume may be partially driven by norovirus activity: more calls were taken during the norovirus season, when a higher proportion of callers were norovirus positive. GII.4 norovirus strains were more prominent during peak norovirus season, and GI and less common GII genotypes were more prominent in the off-season. A review of published norovirus outbreaks found that GII outbreaks were significantly associated with winter seasonality compared with GI outbreaks (*9*). Additionally, GII.4 outbreaks have been associated with severe outcomes, such as hospitalization and death (*10*), underscoring the importance of monitoring their emergence and effects.

The greater proportion of vomiting and fever and longer illness duration among norovirus-positive complainants suggests that a bacterial intoxication, especially with diarrheal toxin agents such as *Clostridium perfringens*, may have caused a substantial proportion of illness among norovirus-negative complainants. However, complainant samples were not routinely tested for bacterial intoxication agents in this study because of the lag time from onset to complaint. Differences in rates of fever and health care visits between eligible complainants and those tested (Table 2) limit the accuracy of extrapolated estimates if these variables affect the likelihood that a caller is norovirus positive. However, if all symptom-eligible complainants are assumed to have the same risk for norovirus infection as the subpopulation of those tested, an estimated 1 in 5 callers during the peak off-season and 3 in 4 callers during the peak season would be infected with norovirus.

These results have limited potential for extrapolation to norovirus incidence estimates for Minnesota. The proportion of the population who would call the hotline when ill is unknown; in addition, hotline callers are not necessarily representative of the general population. However, trends observed among hotline callers, including norovirus prevalence, genotype diversity, and call volume, can serve as indicators of general norovirus activity. For example, our study demonstrates the transition in predominant circulating norovirus strain from GII.4 New Orleans to the emergent GII.4 Sydney strain, as has been observed among US outbreaks (*11*). The emergence of a new GII.4 strain has sometimes been associated with an increase in norovirus outbreak activity (*12*). However, an increase in proportion of callers positive for norovirus during the beginning of the 2012–2013 season was not observed in our study after the emergence of GII.4 Sydney. During this same period, the number of norovirus outbreaks identified by MDH was likewise not higher than in recent years (*12*; MDH, unpub. data), suggesting that GII.4 Sydney did not cause increased norovirus activity in Minnesota. Of note, a complainant with a sporadic case from October 2011 tested through this project was initially identified as being infected with GII.4 New Orleans, but GII.4 Sydney infection was retrospectively identified after CaliciNet updated its reference strains in November 2012 to include GII.4 Sydney.

In conclusion, norovirus accounted for most cases of acute gastroenteritis among hotline callers in Minnesota, particularly during the fall and winter norovirus season. Trends in positive specimens, genotype distribution, and symptom histories observed during complaint-based surveillance can be used to better understand the epidemiology of norovirus gastroenteritis.

Technical Appendix Expanded project methods and data analysis.
