# Supplementary material for: Norovirus Surveillance among Callers to Foodborne Illness Complaint Hotline, Minnesota, USA, 2011–2013
Source: Emerg Infect Dis. 2013 Aug;19(8):1293–6. doi: 10.3201/eid1908.130462 (PMC3739525; doi:10.3201/eid1908.130462)
Supplement: Technical Appendix — Expanded project methods and data analysis. [file 13-0462-Techapp-s1.pdf]

Article DOI: http://dx.doi.org/10.3201/eid1908.130462

Foodborne Illness Complaint Hotline and Norovirus Surveillance, Minnesota, USA, 2011–2013

Technical Appendix

Expanded Project Methods

Study subjects

A Minnesota Department of Health (MDH) staff person administers a standard questionnaire to hotline callers that includes symptom details, timing of onset and recovery, age, a 4-day food history, and healthcare visits, including emergency room, urgent care, primary care, or hospitalization. Symptom information and common meal exposures are also gathered for others reported ill (co-complainants) by the original caller. The complaint system is described in detail by Li et al. (*1*).

From October 1, 2011 through January 31, 2013, eligible hotline complainants were asked to submit a self-collected stool sample to the MDH Public Health Laboratory (PHL) for norovirus testing. Complainants were eligible to submit a stool specimen if they were Minnesota residents, reported experiencing vomiting or diarrhea (≥3 loose stools in 24 hours), and were interviewed ≤4 days from vomiting or diarrhea onset (whichever was earlier) or ≤2 days from vomiting or diarrhea resolution (whichever was later). Only complainants interviewed by MDH staff were asked to submit a stool sample; completed complaint interviews forwarded to MDH from local jurisdictions were not eligible. If the original complainant was not eligible for testing or refused, an eligible co-complainant was asked to submit a stool sample.

Parent or guardian permission was obtained for participants <18 years of age. This project was intended to improve surveillance for norovirus using a pre-existing complaint system and therefore was not classified as research or subject to review by an Institutional Review Board.

Laboratory testing

Stool sample collection kits were sent to up to three co-complainants per complaint; only one stool per complaint received at the MDH PHL was used in analysis. Stool kits included instructions, a Protocult Collection Device (*Ability Building Center, Rochester, Minnesota, USA*) and a Para-Pak C&S Stool Transport Medium sample vial (*Meridian Bioscience, Inc., Cincinnati, Ohio, USA*). On the day of interview and consent to testing, stool kits were hand-delivered to complainant households or sent via Federal Express overnight delivery. Complainants were asked to collect the sample as soon as possible after receipt of the collection kit. Specimens were returned to the MDH PHL in a postage-paid box via regular mail.

At the MDH PHL, specimen vials were refrigerated upon arrival and batch tested weekly. Nucleic acid was extracted using the QIAamp Viral RNA Mini kit (*Qiagen, Valencia, CA, USA*). Detection and characterization of norovirus strains was performed using CDC's CaliciNet methods (*2*). Briefly, detection of norovirus genogroups I and II was performed by duplex real-time reverse transcription polymerase chain reaction (rRT-PCR). Characterization was performed by RT-PCR of the viral capsid gene (Region D and/or Region C) followed by sequence analysis of the PCR product. Genotypes were determined by phylogenetic comparison with CaliciNet reference strains. Sequence results were not uploaded to CaliciNet unless the specimen was associated with an outbreak or requested by CDC.

Participants were informed of their norovirus testing results via telephone. At this time, participants were also asked about dates of symptom resolution. Several attempts were made to reach participants with results.

Data Analysis

Calls from the same complainants at different times during the study period were counted as unique complaints. If a complaint led to identification of a foodborne outbreak, one complaint stool specimen was included in analysis from each outbreak if it met other eligibility criteria.

Based on the known winter seasonality of norovirus outbreaks (*3*), norovirus season was defined as October–March, and the off-season as April–September. The Chi-square test was used to compare categorical variables (sex, diarrhea, vomiting, bloody stools, fever, healthcare visit, and season) between norovirus-positive versus norovirus-negative complainants, and between all

complainants tested versus all symptom-eligible complainants who were not tested. Fisher exact test was used when expected cell frequencies were <5. The nonparametric Wilcoxon two-sample test was used for comparison of medians (age, duration). The two-sample t-test was used for comparison of means. Data analysis was performed using SAS software v9.2 (*SAS Institute Inc., Cary, NC, USA*).

References

1. Li J, Smith K, Kaehler D, Everstine K, Rounds J, Hedberg C. Evaluation of a statewide foodborne illness complaint surveillance system in Minnesota, 2000 through 2006. J Food Prot. 2010;73:2059–64. PubMed

2. Vega E, Barclay L, Gregoricus N, Williams K, Lee D, Vinjé J. Novel surveillance network for norovirus gastroenteritis outbreaks, United States. Emerg Infect Dis. 2011;17:1389–95. PubMed

3. Yen C, Wikswo ME, Lopman BA, Vinje J, Parashar UD, Hall AJ. Impact of an emergent norovirus variant in 2009 on norovirus activity in the United States. Clin Infect Dis. 2011;53:568–71. PubMed http://dx.doi.org/10.1093/cid/cir478
